# Differences in Salivary Alpha-Amylase and Cortisol Responsiveness following Exposure to Electrical Stimulation versus the Trier Social Stress Tests

**DOI:** 10.1371/journal.pone.0039375

**Published:** 2012-07-30

**Authors:** Yoshihiro Maruyama, Aimi Kawano, Shizuko Okamoto, Tomoko Ando, Yoshinobu Ishitobi, Yoshihiro Tanaka, Ayako Inoue, Junko Imanaga, Masayuki Kanehisa, Haruka Higuma, Taiga Ninomiya, Jusen Tsuru, Hiroaki Hanada, Jotaro Akiyoshi

**Affiliations:** Department of Neuropsychiatry, Oita University Faculty of Medicine, Yufu-Shi, Oita, Japan; Max Planck Institute of Psychiatry, Germany

## Abstract

**Background:**

Cortisol is an essential hormone in the regulation of the stress response along the HPA axis, and salivary cortisol has been used as a measure of free circulating cortisol levels. Recently, salivary alpha-amylase (sAA) has also emerged as a novel biomarker for psychosocial stress responsiveness within the sympathetic adrenomedullary (SAM) system.

**Principal Findings:**

We measured sAA and salivary cortisol in healthy volunteers after exposure to the Trier Social Stress Test (TSST) and electric stimulation stress. One hundred forty-nine healthy volunteers participated in this study. All subjects were exposed to both the TSST and electric stimulation stress on separate days. We measured sAA and salivary cortisol levels three times immediately before, immediately after, and 20 min after the stress challenge. The State (STAI-S) and Trait (STAI-T) versions of the Spielberger Anxiety Inventory test and the Profile of Mood State (POMS) tests were administered to participants before the electrical stimulation and TSST protocols. We also measured HF, LF and LF/HF Heart Rate Variability ratio immediately after electrical stimulation and TSST exposure. Following TSST exposure or electrical stimulation, sAA levels displayed a rapid increase and recovery, returning to baseline levels 20 min after the stress challenge. Salivary cortisol responses showed a delayed increase, which remained significantly elevated from baseline levels 20 min after the stress challenge. Analyses revealed no differences between men and women with regard to their sAA response to the challenges (TSST or electric stimulations), while we found significantly higher salivary cortisol responses to the TSST in females. We also found that younger subjects tended to display higher sAA activity. Salivary cortisol levels were significantly correlated with the strength of the applied electrical stimulation.

**Conclusions:**

These preliminary results suggest that the HPA axis (but not the SAM system) may show differential response patterns to distinct kinds of stressors.

## Introduction

Over half a century ago, Selye and McKeown [1] defined “stress” as the body's response when a human being is subjected to an outside stimulus. However, there are many kinds of stressors, including psychosocial, physical, and biological stimuli. To date, no one has proposed a precise index for quantitatively evaluating stress across a variety of stressors.

From studies on human responses to stressful events, neuroendocrine markers play an important role in establishing the bodily reaction to stress. Stress responsiveness is primarily regulated by two neuroendocrine axes: the hypothalamic-pituitary-adrenocortical (HPA) and sympathetic adrenomedullary (SAM) systems [2], [3], [4]. A role for HPA axis activity in mediating stress responses has been intensively investigated for decades. The HPA axis is a complex neuroendocrine stress system involved in bio-behavioral adjustments to confrontational stimuli and change. Cortisol is an essential hormone in the regulation of stress responsiveness. It exists in both free (active) and protein-bound (inactive) forms in serum but only in a free form in saliva. Recently, salivary cortisol has been used as a simple, noninvasive index of free circulating cortisol levels. Salivary cortisol sampling has been used as a measure of HPA axis activity for quite some time [5]. Salivary cortisol levels increase several fold within a short time period after the onset of psychological stress [6] (Stahl and Dorner, 1982) as well as during physical stress such as exercise [7] or cold pressor stress [8].

Recently, much attention has been given to possible interactions between stress and α-amylase levels. However, significant psychosocial studies of α-amylase responsiveness are difficult due to the system's complexities. Salivary α-amylase (sAA) is secreted by the parotid gland in response to adrenergic activity and is suppressed by β-adrenoreceptor blockade [9]. It has also become established as a new biomarker of the psychosocial stress response within the SAM system [10]. Studies show marked increases in sAA levels in response to stressful tasks or procedures, such as a parachute jump [11] or a stressful video game [12], as well as to other types of psychological (e.g. pre-examination) stressors [13], [14]. Stimulation of sAA levels by psychosocial stress was demonstrated in studies employing the Trier Social Stress Test (TSST) [15], [16], [17] and in experiments in which subjects underwent a stressful fMRI procedure involving negative emotional picture viewing [18] or were exposed to video-based stressors [19]. Finally, pharmacological manipulation of the SAM system has underscored the role of sAA amylase as an indicator of sympathetic activity. Stimulation of the SAM system by administration of yohimbine (an alpha-2 adrenergic receptor antagonist) was shown to significantly increase sAA levels [20].

Activation of the stress response in reaction to a threatening, negative, or unexpected experience evokes a chain of neuroendocrine and other nervous system reactions. The SAM system, via catecholamines (noradrenaline and adrenaline) signaling and interacting with glucocorticoids (HPA system), plays a key role in both normal homeostasis and in sympathetically mediated responses to stress [21]. The various roles of glucocorticoids (GCs) in stress responsiveness have been extensively reviewed [22]. GC actions permit, stimulate, or suppress an ongoing stress response, -or can even be preparative for a subsequent stressor. However, the exact manner in which these systems interact to mediate the stress response is equivocal. Although many studies looked at either noradrenaline or cortisol responses in reaction to stress, overwhelming evidence suggests that both systems are part of a coherent unity that requires concerted action. More studies are needed to look at the way these systems interact, especially when both systems are activated [8]. Some studies have investigated the stress response to pain [23], [24], [25], [26], [27]. However, most reports are conducted in patients with trauma, chronic pain, or psychiatric disorders including anxiety disorder. It is unknown exactly how pain-causing stressors interact within these two stress-response systems, especially in healthy subjects. Furthermore, to our knowledge, there are few studies comparing the SAM system with the HPA system in response to two different stress domains such as psychological and physical stress [8]. Recently, sAA and salivary cortisol stress response patterns were described at various time points throughout a stressful situation [21].

The current study was designed to investigate the responses of the two primary neuroendocrine systems (the HPA and SAM systems) to two different stressors. To evaluate the effects of different stressors on the HPA and SAM systems, we assessed the secretion of sAA and salivary cortisol in healthy volunteers after exposure to a psychosocial stress task (the Trier Social Stress Test; TSST) and a physical stress task (electrical stimulation, a pain-causing stress).

The aim of the present study was twofold. The first research question was whether sAA and salivary cortisol responses were related during the two different stress tasks. We hypothesized that SAM system and HPA axis responses on these tasks are interconnected. More precisely, we hypothesized that subjects reacting with sAA increases on one task would also increase salivary cortisol levels on the same task, and that they would be more sensitive to stressful stimuli, setting up the two systems for a stronger response to the other task. Therefore, we hypothesized that a strong sAA or salivary cortisol response on one task predicts a stronger sAA and salivary cortisol response on the other stress task. The second research question referred to the hypothesis that men and women might differ in their stress responsiveness. Several studies have shown that men and women differ in their response to stressful events in terms of their personal emotional rating of emotional material (women almost always rating higher than men) or their emotional memory performance [28], [29], [30], [31]. Several studies found a stronger salivary cortisol response in men versus women in reaction to stressors [32], [33]. However, only a few studies have focused on sex differences in sAA levels or responsiveness and these studies produced ambiguous results [34], [35], [36]. Therefore, we wanted to explore whether gender differences affected baseline sAA and salivary cortisol levels as well as the reactivity of and the interaction between both hormonal systems.

## Materials and Methods

### Subjects

**Figure 1:**
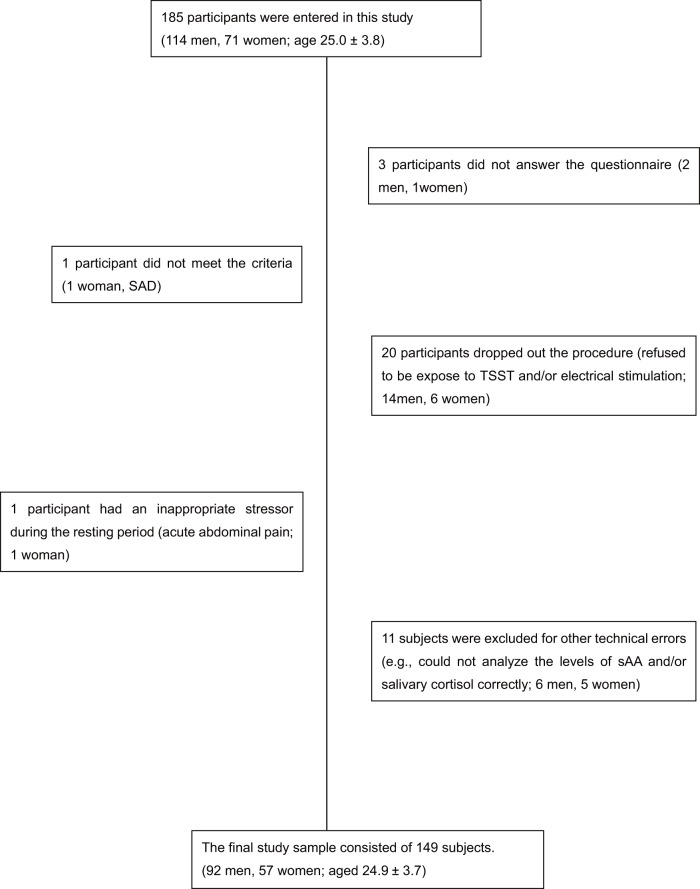
Flow chart in the present study. SAD; Social Anxiety Disorder. TSST; Trier Social Stress Test. sAA; salivary amylase.

One hundred and eighty-five healthy volunteers with no history of psychiatric disorder were recruited from the Oita University Hospital staff (114 men and 71 women, aged 25.0±3.8 years, range 22–45 years) and participated in this study. All participants were not medicated at the time of testing. Exclusion criteria were comprised of the following: psychiatric disorders, distinct physical or toxic disorders, a body mass index of 32 or greater, use of steroid-based medications within the past three years, pregnancy, drug/alcohol abuse, and current tobacco use. All participants had Beck Depression Inventory 2 (BDI-2; Beck et al., 1974) scores of 7 or less, and had no history of depression. Further diagnostic exclusion criteria included: any other mental disorders according to the Mini-International Neuropsychiatric Interview (MINI) and any acute and/ or chronic medical illness. Three participants did not answer the questionnaires (2 men, 1 women), one participant did not meet the inclusion criteria (1 woman; SAD), twenty participants dropped out during the procedure (refused to be exposed to TSST and/or electric stimulation; 14 men, 6 women), one participant had an inappropriate stressor during the resting period (acute abdominal pain; 1 woman), and eleven subjects were excluded for other technical errors (e.g., could not analyze the levels of sAA and/or salivary cortisol correctly; 6 men, 5 women). All subjects were exposed to both the electrical stimulation stress and the Trier Social Stress Test (TSST; 5-min speech task plus 5-min mental arithmetic task) at random in a counterbalanced order over one week. The final study sample consisted of 149 subjects (92 men and 57 women, aged 24.9±3.7 years, range 22–45 years; study flow chart is shown in Figure 1). Subjects included 137 Oita University Students, 7 clerks, and 5 doctors, all of whom were college educated. Written informed consent was obtained from all participants following a description of the procedures and risks, and participants had the opportunity to ask questions. This study was approved by the Ethics Committee of Oita University.

### Procedures

All subjects were exposed to both TSST and electric stimulation stress on separate days during 1 week. To exclude the effect of being habituated and relaxed to the experimental environment, participants were divided into small groups consisting of four to five subjects. Each group was assigned to the first trial's experiment of electric stimulation or TSST, alternately. The second trial's experiment was administered one week after the first experiment. The subjects were told to refrain from taking any medication for five hours before arrival and to refrain from brushing their teeth or eating for at least 60 min before the measurement session. To examine sAA and salivary cortisol stress responses, we measured sAA and salivary cortisol levels three times (immediately before, immediately after, and 20 min after the intervention) in both experiments consistent with previous reports [27], [37]. We also examined the interaction between sAA levels, salivary cortisol levels, and the different stress challenges. To evaluate the responsiveness over time, the area under the curve with respect to increase (AUCi) was calculated for both sAA and salivary cortisol levels according to Pruessner et al. [38]. Furthermore, a delta-increase was also calculated for each biomarker according to Strahler et al. [39]. To control for circadian variations in sAA and salivary cortisol levels, the exposure to physical stressors and psychological stressors and collection of saliva were performed between 13∶00 and 16∶00 h [40]. To exclude the effect of thermal stress [41], the experimental setting was kept at a room temperature of 25±2°C throughout the study protocol. Salivary α-amylase (sAA) was measured using the Dry Chemistry System (Nypro Corp., Japan) according to the manufacturer's protocol. This measurement method using a hand-held monitor can be performed easily and quickly and is a convenient and useful objective indicator for medical and educational practice [42]. The hand-held monitor consisted of a disposable test strip and a monitor [42], [43]. This method for analyzing sAA has previously been evaluated and employed [43], [44], [45]. Saliva was sampled by directly immersing a saliva-sampling strip in saliva under the tongue for 30 s [46], [47]. The strip was immediately placed in an automatic saliva transfer system and saliva was transformed to the α-amylase test paper on the reverse side of the strip sleeve by compression. The α-amylase test paper contained the substrate 2-chloro-4-nitrophenyl-4-O-β-D-galactopyranosylmaltoside (Gal-G2-CNP). The enzyme reaction started upon transfer by compression and the free CNP level was optically measured after 20 s. The alpha-amylase activity that reduced sugars equivalent to 1 μmol/min of maltose was defined as 1 unit. The concentration of salivary cortisol was analyzed by ELISA assay [48], with intra-assay and inter-assay coefficients of variation of 3% and 10%, respectively. Samples were stored at −20°C until thawed for analysis.

We administered the State (STAI-S) and Trait (STAI-T) versions of the Spielberger Anxiety Inventory [49] tests to participants before electrical stimulation and TSST protocols. We also administered the Profile of Mood State (POMS) test to participants before the electrical stimulation and TSST protocols. Finally, we also examined HF, LF and LF/HF Heart Rate Variability (HRV) ratio immediately after electrical stimulation and TSST by using an APG Heart-Rater SA 3000P (Tokyo Iken Co, Ltd, Japan).

### Electrical stimulation

**Figure 2:**
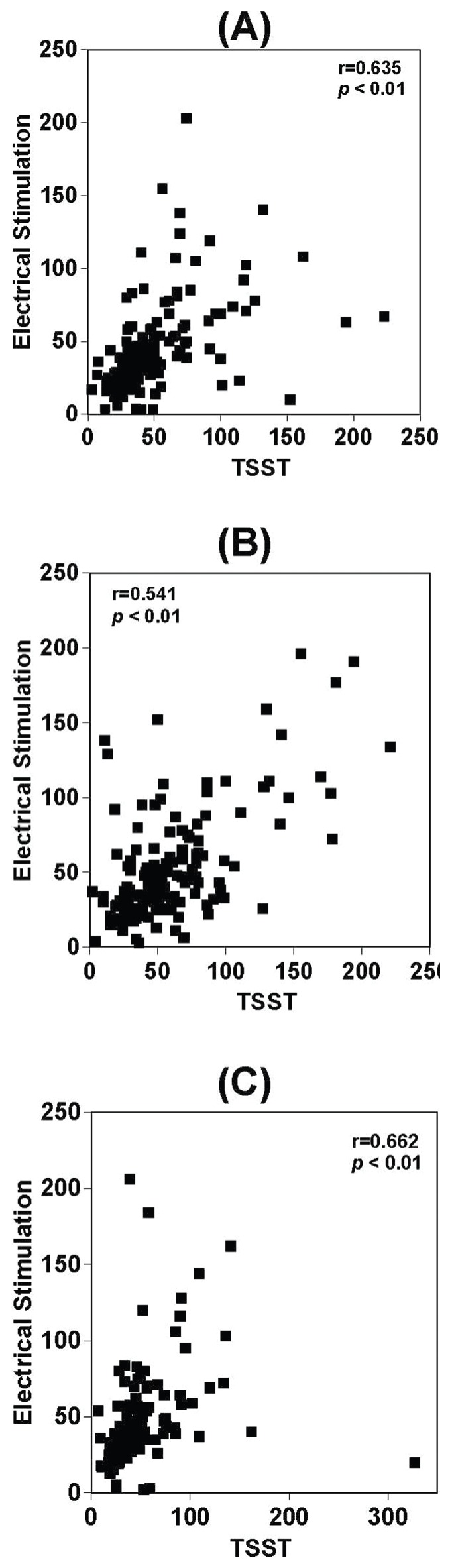
Relationship between sAA, TSST and electrical stimulation. Relationship of basal sAA levels (A), sAA levels after electrical stimulation (B) and sAA levels 20 min after electrical stimulation (C) between TSST and electrical stimulation exposure.

All participants were invited to our laboratory on a weekday between 13∶00 and 16∶00 in the afternoon (Figure S1). Subjects wore stimulator coils connected to a stimulator on the wrist. This device provided electrical current to the motor and sensory fibers of the median nerve in the right wrist [27]. Subjects were stimulated in incremental steps until they reached their threshold stimulus, defined as the greatest stimulus they could tolerate. The threshold stimulus for each subject was applied for 40 seconds. The mean amplitude of electrical stimulation was between 2–55 mA. Thus, the magnitude of electrical stimulation experimentally varied across subjects. Subjects were told that the level of electrical stimulation would be sufficient to cause pain but would not cause burning or other injury. Electric stimulation lasted only briefly and caused no physical impairment [27]. The amplitude applied appeared to reflect individual sensitivities. The threshold of electrical stimulation might be dependent on inter-individual differences related to psychological factors such as depression and anxiety [27], [50]. Moreover, the strength of the stimulation was determined by each subject. Therefore, this pain-causing experimental setting was not contrary to the ethics of human experimentation. The experimental timeline is shown in Figure 2.

### Trier Social Stress Test

**Figure 3:**
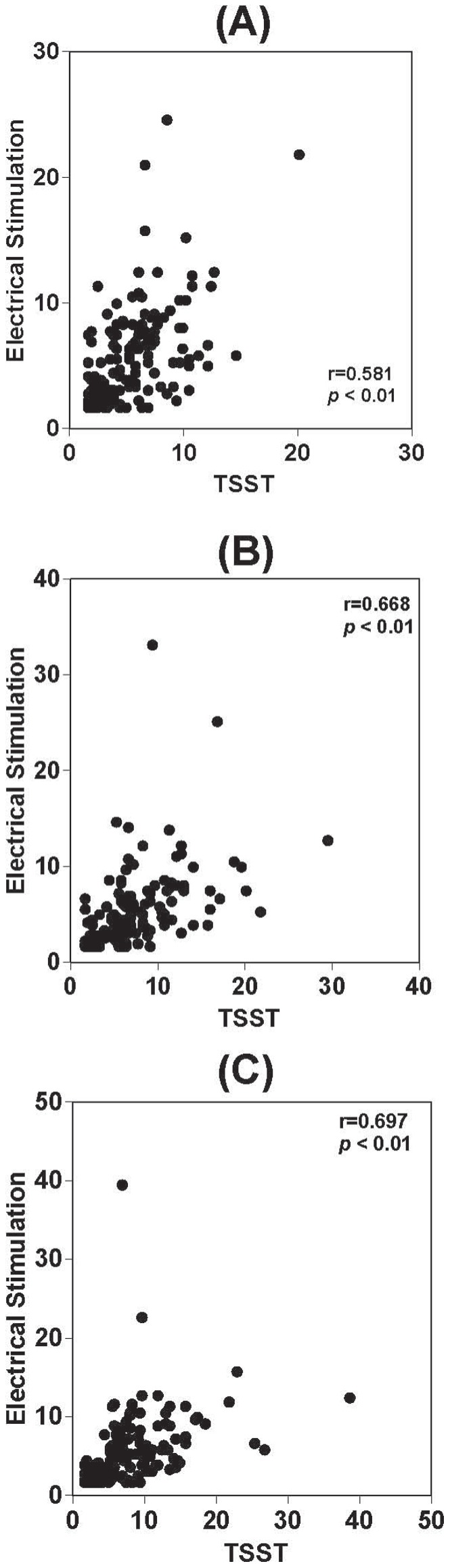
Relationship between salivary cortisol, TSST and electrical stimulation. Relationship of basal salivary cortisol levels (A), salivary cortisol levels after electrical stimulation (B) and salivary cortisol levels 20 min after stimulation (C) between TSST and electrical stimulation exposure.

All participants were invited to our laboratory on a weekday between 13∶00 and 16∶00 in the afternoon. After a 30-min resting period to minimize the impact of physical activity, prior stress, and emotions, during which all participants filled in some questionnaires, participants were exposed to the Trier Social Stress Test (TSST) [51]. The TSST consists of a 3-min preparation period, a 5-min speech task, during which participants have to discourse about their personal characteristics, followed by a 5-min mental arithmetic task, both in front of an audience (Figure S2). After this stress task, participants remained in our laboratory for another 20 min for a collection of samples during recovery. The experimental timeline is shown in Figure 3.

### Statistical analysis

As the data were not normalized (log transformed), we employed nonparametric statistics. Pearson's correlation was used to investigate the relationship between the questionnaire results and the subjects' actual neuroendocrine reaction. The results of the questionnaire (STAI and POMS) were analyzed using Mann Whitney-*U* tests due to the ordinal level of measurement. Where sAA and salivary cortisol levels showed a skewed distribution, these data were normalized by natural log transformation.

Spearman's rank correlation coefficient tests were used to examine the correlation between AUCi or delta-increase for each biomarker and the strength of electrical stimulation applied. sAA and salivary cortisol data were analyzed using 2 (sex; male/female) × 2 (condition: electrical stimulation/TSST) ×2 (time: baseline/immediately/after stimulation/20 min after stimulation) repeated-measures ANOVAs. *Post hoc* analyses using the least significant difference (LSD) method were conducted to determine subgroup differences. Finally, multiple regression analysis were used to predict the two different indices for each biomarker response (AUCi and delta-increase) by indices for sAA, salivary cortisol, age, HF, LF & LF/HF, STAI-S & STAI-T scores, and POMS scores. The results are expressed as means ± SD of the individual values from each test. Statistical significance was set at *p*<0.05.

To evaluate the *a posteriori* effect size for the outcome of each response, Cohen's *f* was calculated using G*Power version 3.1.2 [52]. Cohen's *f*-values were interpreted using the following criteria: 0.1 to 0.24 represents a small effect size, 0.25 to 0.39 represents a medium effect size, and greater than 0.4 represents a large effect size.

## Results

**Figure 4:**
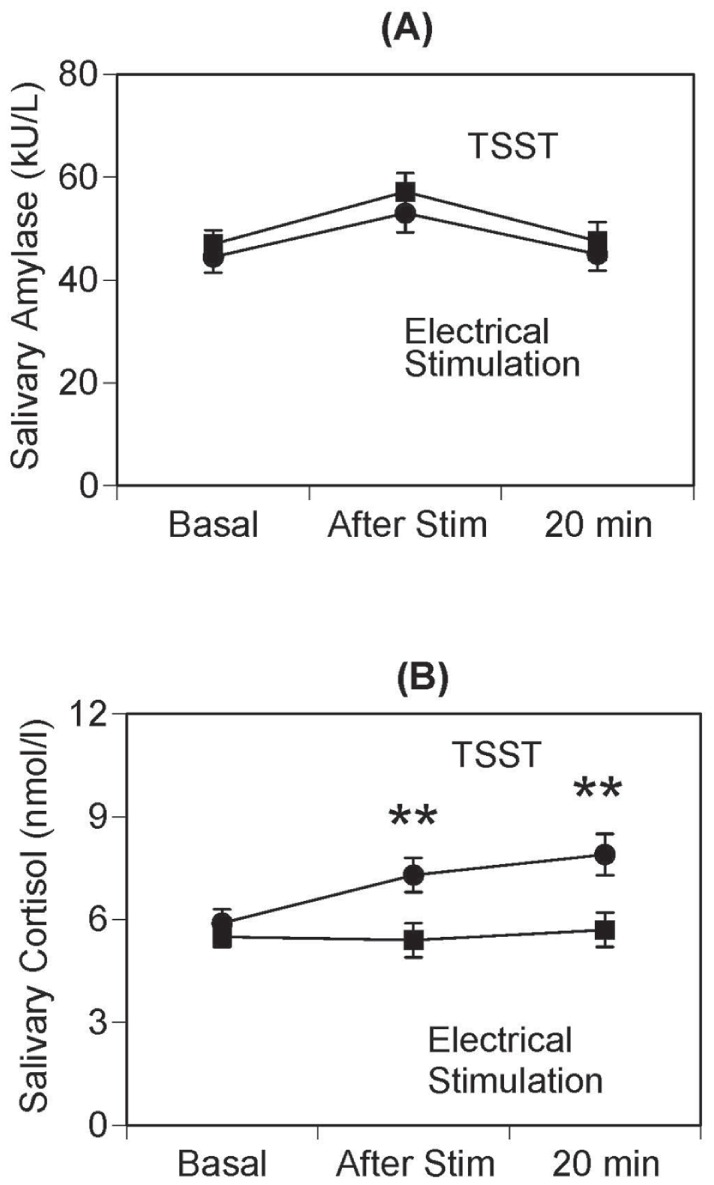
sAA responsiveness between TSST and electrical stimulation. There was no difference in sAA responsiveness between TSST and electrical stimulation challenges (A). In comparison, salivary cortisol responsiveness following TSST exposure was significantly enhanced compared to after electrical stimulation exposure. Values are presented as mean ± standard deviation. ** *p*<0.01.

There was no significant difference in sAA responsiveness between electric stimulation and TSST exposure (*F*(1, 297) = 0.44, p>0.05) (Figure 4A). A notable sAA response to the interventions was found with peak sAA values registered immediately after the interventions. We found no significant sAA response to electrical stimulation (Figure 4A). There were no sex differences in terms of sAA response to electrical stimulation and TSST (*F*(1, 297) = 0.50, p = 0.48; *F*(1, 297) = 0.72, p = 0.40). The salivary cortisol response to the TSST was significantly increased more than the salivary cortisol response to electrical stimulation (*F*(1, 297) = 6.54, *p*<0.05) (Figure 4B). In addition, salivary cortisol displayed a prolonged responsiveness after TSST. There were significant salivary cortisol level increases between baseline and immediately after stimulation (*p*<0.01), and between immediately after stimulation and 20 minutes later. Gender differences in the response to stress, and especially in the reactivity to stress, may provide cues to explain the greater rates of depression in females [53]. Some reports suggest that there are no gender differences in the relationship between sAA levels and stress [54], [55], [56], while others suggest that men have higher sAA levels versus women during stressful tasks [8]. Further studies are needed to examine the relationship between stress and both gender.

There was a significantly relationship between Tension-Anxiety scores on POMS and salivary cortisol levels after electrical stimulation. There was no relationship between other POMS scores and salivary cortisol levels after electrical stimulation. There was no relationship between any POMS score and sAA levels after electrical stimulation. There was no relationship between any POMS score and sAA levels following TSST exposure. There was no relationship between any POMS score and salivary cortisol levels after TSST exposure. There was no relationship between STAI scores and sAA or salivary cortisol levels after electrical stimulation. There was no relationship between STAI scores and sAA or salivary cortisol levels following TSST exposure. There was no relationship between HRV variables and sAA or salivary cortisol levels after either electrical stimulation or TSST exposure. Some reports suggest a negative relationship between salivary cortisol concentrations and anxiety scores in adolescents [57], although an analysis of changes in cortisol levels revealed a non-linear interactive effect between stress/anxiety and time of day [58].

We found no significant salivary cortisol response to electrical stimulation (Figure 4B). There were no sex differences in terms of salivary cortisol response to electrical stimulation or TSST (*F*(1, 297) = 0.21, p = 0.65; *F*(1, 297) = 0.06, p = 0.80). Calculated effect sizes for the outcome of each response were 0.19. The Type I error α was 0.04 and Power (1-β) was 0.80.

**Figure 5:**
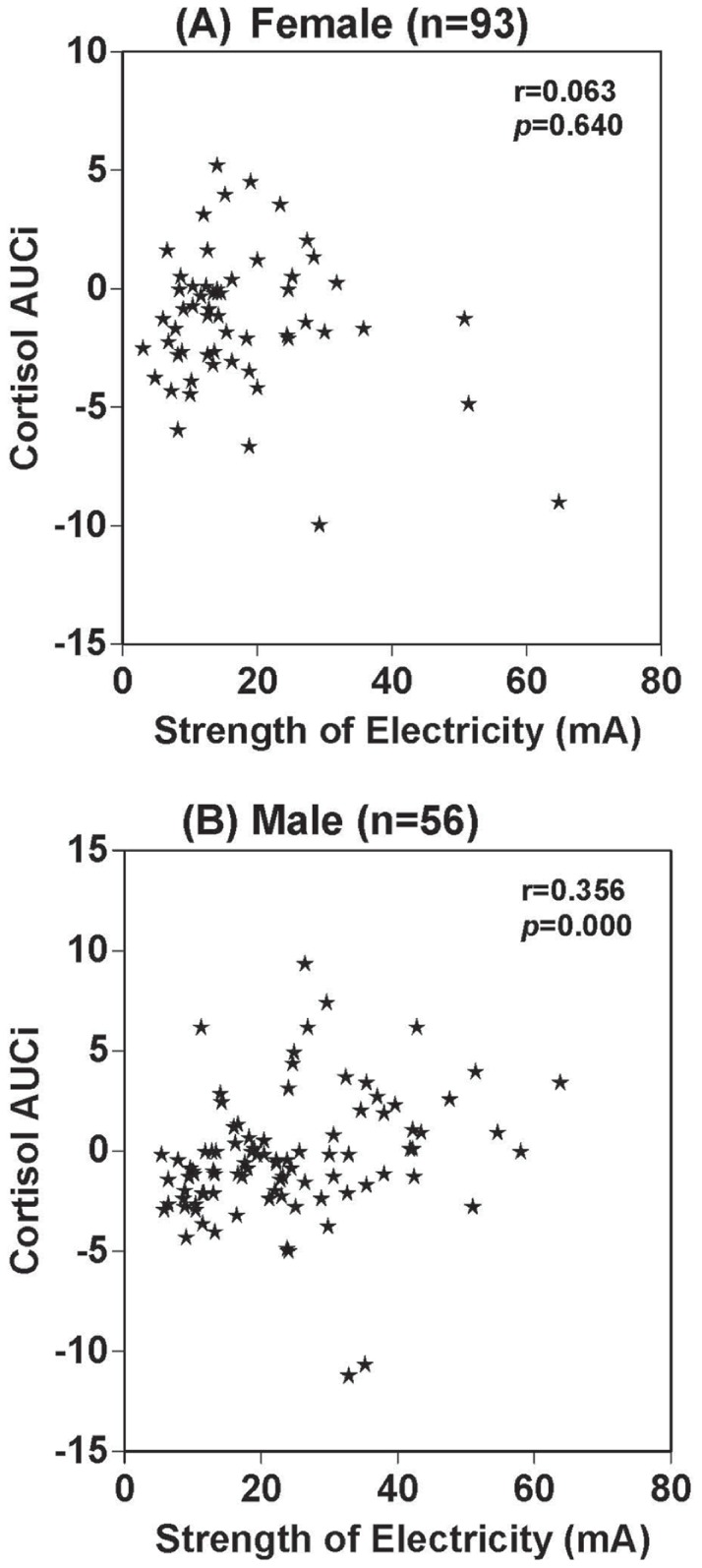
Correlation between sAA, salivary cortisol AUCi and electrical current. Correlation between sAA AUCi and strength of applied electrical current (A). Correlation between salivary cortisol AUCi and strength of applied electrical current (B).

**Figure 6:**
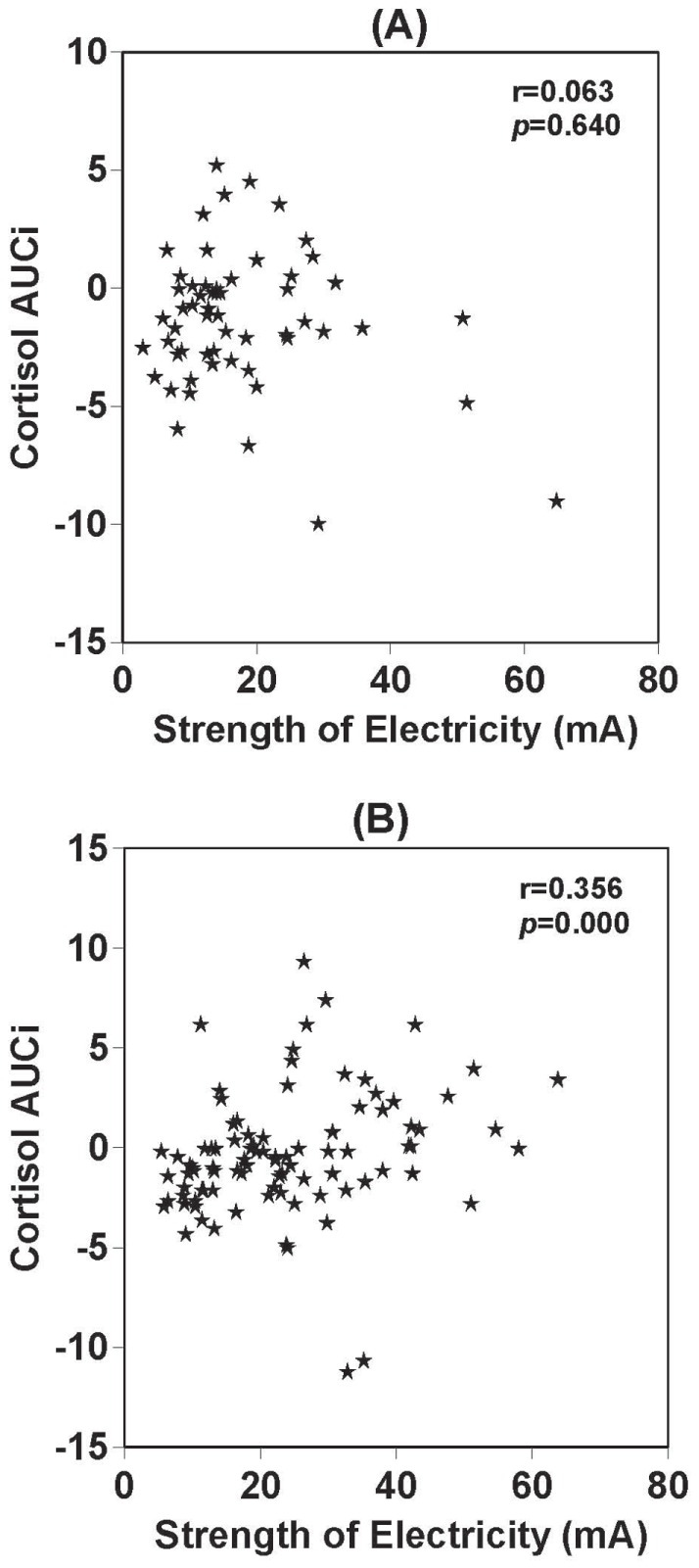
Correlation between salivary cortisol AUCi and electrical current in females or males. Correlation between salivary cortisol AUCi and strength of applied electrical current in females (n = 93) (A). Correlation between salivary cortisol AUCi and strength of applied electrical current in males (n = 56) (B).

There was a significant relationship between TSST exposure and electrical stimulation in terms of basal sAA levels (r = 0.635, p<0.01), sAA levels after electrical stimulation (r = 0.541, p<0.01) and sAA levels 20 min after stimulation (r = 0.662, p<0.01) (Figure 2. A, B, C). There was also a significant relationship between TSST exposure and electrical stimulation in terms of basal salivary cortisol levels (r = 0.581, p<0.01), salivary cortisol levels after electrical stimulation (r = 0.688, p<0.01) and salivary cortisol levels 20 min after electrical stimulation (r = 0.697, p<0.01) (Fig. 3. A, B, C). There were no sex differences in terms of salivary cortisol response to electrical stimulation or TSST. sAA AUCi was not correlated with applied strength of electrical current (r = 0.074, p>0.05) (Figure 5A). In contrast, salivary cortisol AUCi was significantly correlated with the strength of electricity applied (r = 0.252, p<0.01) (Figure 5B). Salivary cortisol AUCi was not correlated with the strength of electricity applied in females (r = 0.063, p = 0.640) (Figure 6A), but was correlated in males (r = 0.356, p = 0.000) (Figure 6B). Judging from the correlations shown in Figures 4 and 5 (A and B panels), one might infer that the magnitude of electrical stimulation was experimentally varied across subjects. However, the values along the axes reflect the large inter-individual variation of pain thresholds in response to electrical stimulation.

As indicated in Table 1, there was no relationship between Tension-Anxiety (r = −0.105, p = 0.202), Depression-Dejection (r = −0.123, p = 0.134), Anger-Hostility (r = −0.137, p = 0.097), Vigor (r = −0.007, p = 0.932), Fatigue (r = −0.04, p = 0.599), or Confusion (r = −0.098, p = 0.237) scores on the POMS test and sAA levels before electrical stimulation. There was a significant relationship between Tension-Anxiety (r = −0.174, p = 0.034) scores on the POMS test and salivary cortisol levels before electrical stimulation. There was no relationship between Depression-Dejection (r = −0.146, p = 0.075), Anger-Hostility (r = −0.102, p = 0.215), Vigor (r = −0.028, p = 0.737), Fatigue (r = −0.008, p = 0.922), or Confusion (r = −0.003, p = 0.966) scores on the POMS and salivary cortisol levels before electrical stimulation. There was also no relationship between Tension-Anxiety (r = −0.026, p = 0.751), Depression-Dejection (r = −0.044, p = 0.596), Anger-Hostility (r = −0.054, p = 0.514), Vigor (r = −0–0.003, p = 0.973), Fatigue (r = −0.009, p = 0.913), or Confusion (r = −0.054, p = 0.513) scores on the POMS test and sAA levels before the TSST. There was also no relationship between Tension-Anxiety (r = 0.037, p = 0.652), Depression-Dejection (r = 0.018, p = 0.826), Anger-Hostility (r = 0.081, p = 0.324), Vigor (r = −0.050, p = 0.542), Fatigue (r = −0.006, p = 0.946), or Confusion (r = 0.036, p = 0.661) scores on the POMS test and salivary cortisol levels before the TSST.

**Table 1:** Characteristics of electrical stimulation and TSST.

Psychological Test	Electrical Stimulation (n = 149)				TSST (n = 149)			
	Salivary Amylase	*p*	Salivary Cortisol	*p*	Salivary Amylase	*p*	Salivary Cortisol	*p*
**POMS**								
**Tension-Anxiety**	−0.105	0.202	**−0.174**	**0.034**	−0.260	0.751	0.037	0.652
**Depression-Dejection**	−0.123	0.134	−0.146	0.075	−0.044	0.596	0.018	0.826
**Anger-Hostility**	−0.137	0.097	−0.102	0.215	−0.054	0.514	0.081	0.324
**Vigor**	−0.007	0.932	−0.028	0.737	−0.003	0.973	−0.050	0.542
**Fatigue**	−0.040	0.599	−0.008	0.922	−0.009	0.913	−0.006	0.946
**Confusion**	−0.098	0.237	−0.003	0.966	−0.054	0.513	0.036	0.661
**STAI**								
**Trait Anxiety**	−0.032	0.703	−0.075	0.364	−0.016	0.843	−0.076	0.359
**State Anxiety**	−0.025	0.765	−0.099	0.232	0.019	0.823	−0.041	0.620
**Heart Rate Valiability**								
**LF**	−0.094	0.251	−0.055	0.500	−0.030	0.712	−0.112	0.172
**HF**	0.094	0.251	0.055	0.499	0.030	0.713	0.112	0.172
**LF/HF**	−0.094	0.251	−0.057	0.488	−0.030	0.712	−0.112	0.171

Spearman's rank correlation coefficients were used to statistically compare the linear correlations between two tests (electric stimulation and TSST). p-value <0.05 were indicated in bold.

There was no relationship between STAI-S (r = −0.025, p = 0.765) or STAI-T (r = −0.032, p = 0.703) scores and sAA levels after electrical stimulation. There was no relationship between STAI-S (r = −0.099, p = 0.232) or STAI-T (r = −0.075, p = 0.364) scores and salivary cortisol levels after electrical stimulation. There was no relationship between STAI-S (r = 0.019, p = 0.823) or STAI-T (r = −0.016, p = 0.843) scores and sAA levels after exposure to the TSST. There was no relationship between STAI-S (r = −0.041, p = 0.620) or STAI-T (r = −0.076, p = 0.359) scores and salivary cortisol levels following the TSST (see Table 1).

There was no relationship between sAA AUCi and LF (r = −0.094, p = 0.251), HF (r = 0.094, p = 0.251), or LF/HF ratio (r = −0.094, p = 0.251) after electrical stimulation. There was also no relationship between salivary cortisol AUCi and LF (r = −0.055, p = 0.500), HF (r = 0.055, p = 0.499), or LF/HF ratio (r = −0.057, p = 0.488) after electrical stimulation. There was no relationship between sAA AUCi and LF (r = −0.030, p = 0.712), HF (r = 0.030, p = 0.713), or LF/HF ratio (r = −0.030, p = 0.171) following the TSST. There was also no relationship between salivary cortisol AUCi and LF (r = 0.112, p = 0.172), HF (r = 0.112, p = 0.172), or LF/HF ratio (r = −0.030, p = 0.171) following the TSST (see Table 1).

## Discussion

We examined stress responses after physical (electric stimulation) and psychosocial stressors (the Trier Social Stress Test) in the same subjects. There was no significant correlation between the stress response (AUCi of sAA or salivary cortisol) and STAI or POMS subscores. Both after electric stimulation and after the Trier Social Stress Test, we found a rapid response in sAA reactivity, displaying peak levels immediately after the intervention, and recovering to pre-intervention levels 20 min after the interventions. Comparatively, salivary cortisol showed a delayed responsiveness that continued to increase 20 min after the Trier Social Stress Test, but not after electric stimulation.

The original studies on the physiology of the stress response by Walter Cannon and Hans Selye suggested the body's reaction is nonspecific in nature, and thus that all stressors in general produce the same ends. Canon developed the concept of homeostasis and stress by postulating that stress disturbs equilibrium, and that the autonomic response helps to restore one's internal processes to steady-state levels necessary for health and survival in the face of challenge [58]. Selye [59] expanded upon Cannon's work by investigating the other primary system involved in stress: the HPA axis. Selye focused on the release of hormones (glucocorticoids, GCs) from the adrenal cortex and their role in the stress response. He coined the concept of a General Adaptation Syndrome (GAS), which represents a reliable pattern of physiological reactions that correspond to the body's attempt to mediate resistance to a threat. However, our results show that different stressors induce the different stress response. Specifically, two different stressors did not produce the same ends.

Physiological stress, such as electrical stimulation stress is regulated via activation of the HPA and SAM axes. In the present study, electrical stimulation increased sAA response, but not the salivary cortisol response. The difference between sAA and salivary cortisol responses after electric stimulation might be dependent on the latency to reach peak levels of sAA and salivary cortisol. The latency to reach peak levels might be longer for cortisol than for amylase, and a delayed onset might be observed with the cortisol response, but not in regard to amylase levels. TSS exposure increased sAA and salivary cortisol responses. There were significant differences in salivary cortisol responsiveness between electric stimulation and TSS exposure. Namely electrical stimulation and TSST produced the same response pattern in terms of sAA levels, but did not produce different patterns in salivary cortisol release. These results suggest that sAA and salivary cortisol might have a different role in stress physiology. The sAA response may be more sensitive relative to the salivary cortisol response to physiological stressors such as electric stimulation. The differences in amylase and cortisol responsiveness over time may be caused by the differences in the two stress response systems. The SAM system responds via hormonal reaction, whereas the HPA system responds via neuronal reaction. People often report an aversive state resulting from the stress of another, but this could be conveyed through resonating arousal or distress, without activating the physiological stress response [60]. Physiological stress is particularly important to examine since it commonly occur chronically, with known negative effects on health. Physiological stress also includes a general arousal component that is associated with reactivity of the SAM axis and is measured via autonomic nervous system indices such as sAA [61].

We found no significant sex differences in sAA responses after electric stimulation. We also found no significant sex differences in salivary cortisol responses after electric stimulation. Salivary cortisol AUCi was significantly correlated with the strength of applied current in males, but not in females. Our results are consistent with studies reporting that stress exposure or elevated/rising cortisol levels are associated with enhanced fear conditioning in males, but not females [62], [63], [64].

This study has four main limitations. One is that the number of subjects used was relatively small, and we will increase the number of participants in future studies. The second limitation is that the number of hormonal examinations was limited, and we have to increase the number of hormonal responses tested. A third is that we used the time period immediately prior to the stressor as the baseline, and sAA and salivary cortisol levels might differ according to the length of time a participant had been in the hospital [65]. In the future studies, we will try to examine the relationship between endocrinologic responses, neuroimaging data (fMRI, PET and NIRS), and immunological reactions.

In conclusion, our study indicates that the responses of the HPA and the SAM systems may differ depending on sex or the type of stressor. The SAM system, which is the neuronal response, may react more sensitivity to pain-causing stressor (electric stimulation) than the HPA system, which is hormonal response. These preliminary results suggest that the HPA axis (but not the SAM sytem) may show differential response patterns to distinct kinds of stressors.

## Supporting Information

Figure S1 **Experimental timelines after electrical stimulation.** Subjects wore stimulator coils connected to a stimulator on the wrist. This device provided electrical current to the motor and sensory fibers of the median nerve in the right wrist. Subjects were stimulated in incremental steps until they reached their threshold stimulus, defined as the greatest stimulus they could tolerate.
(JPG)

Figure S2 **Experimental timelines following the Trier Social Stress Test.** The TSST consists of a 3-min preparation period, a 5-min speech task, during which participants have to discourse about their personal characteristics, followed by a 5-min mental arithmetic task, both in front of an audience.
(JPG)
