# Supplementary figures and images for: Differences in Salivary Alpha-Amylase and Cortisol Responsiveness following Exposure to Electrical Stimulation versus the Trier Social Stress Tests
Source: PLoS One. 2012 Jul 30;7(7):e39375. doi: 10.1371/journal.pone.0039375 (PMC3408464; doi:10.1371/journal.pone.0039375)

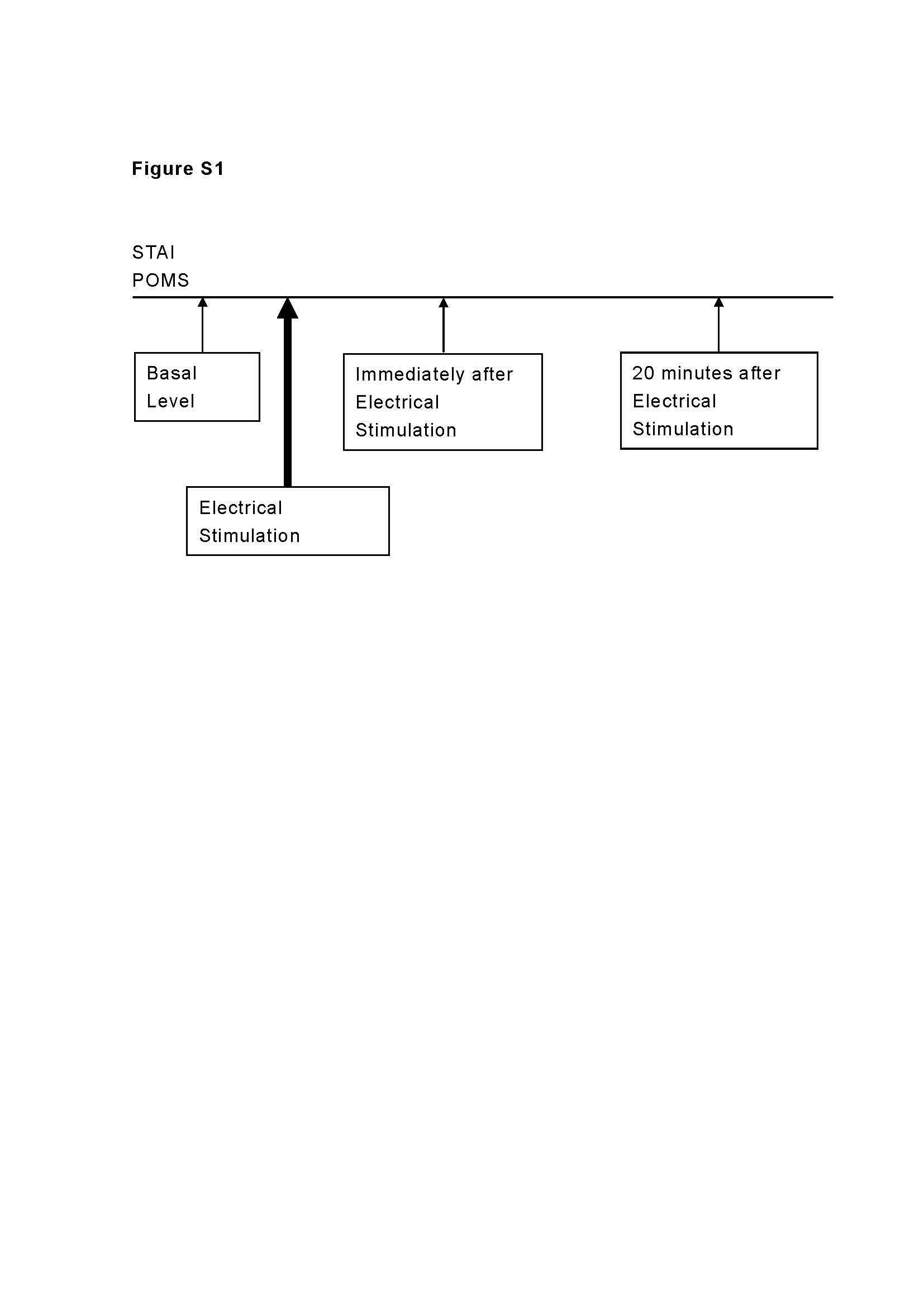

Supplement: Figure S1 — Experimental timelines after electrical stimulation. Subjects wore stimulator coils connected to a stimulator on the wrist. This device provided electrical current to the motor and sensory fibers of the median nerve in the right wrist. Subjects were stimulated in incremental steps until they reached their threshold stimulus, defined as the greatest stimulus they could tolerate. (JPG) [file pone.0039375.s001.jpg]

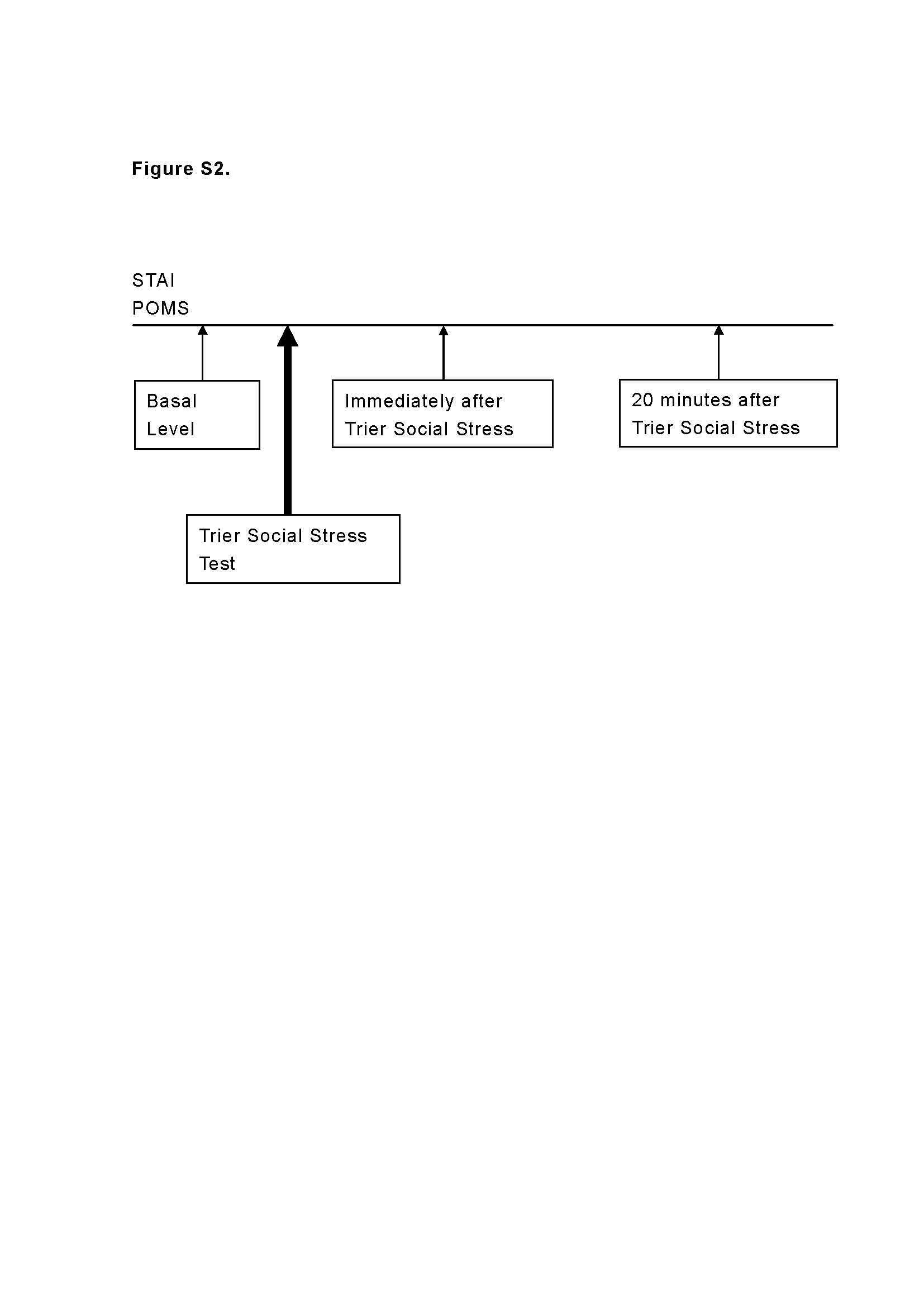

Supplement: Figure S2 — Experimental timelines following the Trier Social Stress Test. The TSST consists of a 3-min preparation period, a 5-min speech task, during which participants have to discourse about their personal characteristics, followed by a 5-min mental arithmetic task, both in front of an audience. (JPG) [file pone.0039375.s002.jpg]
